# Thompson loop: opportunities for antitubercular drug design by targeting the weak spot in demethylmenaquinone methyltransferase protein†

**DOI:** 10.1039/d0ra03206a

**Published:** 2020-06-19

**Authors:** Adeniyi T. Adewumi, Opeyemi S. Soremekun, Mary B. Ajadi, Mahmoud E. S. Soliman

**Affiliations:** Molecular Bio-computation and Drug Design Laboratory, School of Health Sciences, University of KwaZulu-Natal Westville Campus Durban 4001 South Africa soliman@ukzn.ac.za +27 31 260 7872 +27 31 260 8048; Department of Medical Biochemistry, School of Laboratory Medicine and Medical Sciences, College of Health Sciences, University of KwaZulu-Natal Howard Campus Durban 4000 South Africa

## Abstract

Drug-resistant Tuberculosis (TB) has remained the top global health challenge, with a yearly estimation of 10 million infections and 1.5 million deaths in humans. Demethylmenaquinone methyltransferase (*menG*) catalyzes demethylmenaquinone conversion to menaquinone (MK) that is implicated in the TB pathogenesis, hence, it has become a major drug target. DG70 is a biphenyl amide compound known to be a high binding affinity inhibitor of *menG*. This study investigated the structural and dynamic impacts of DG70 upon binding to *menG* using atom-based dynamic simulation. Our findings revealed that the modeled structure of *menG* possesses some Rossman-like methyltransferase characteristic features including two GXG motifs, an omega-like loop (residues 210–220) called the Thompson loop, nine α-helices, five β-strands, *etc.* Furthermore, atom-based dynamic simulations revealed that the Thompson loop is critical in the therapeutic activity of DG70. The loop assumed an open conformation in the unliganded-*menG* structure. However, in the DG70-*menG*, it assumed a tightly closed conformation. This explains the high binding affinity (−32.48 kcal mol^−1^) observed in the energy calculations. Interestingly, these findings are further collaborated by the conformational perturbation in the *menG* protein. Conclusively, insights from this study, highlight the structural “Achilles heel” in *menG* protein which can be further leveraged by inhibitors tailored to specifically target them.

## Abstract 2

Graphical superimposed snapshots of the Thompson novel loop (yellow) of *menG* protein: apo (A) and bound (B) systems. The loop switches between open and closed conformations; critical for therapeutic activity.

## Graphical abstract

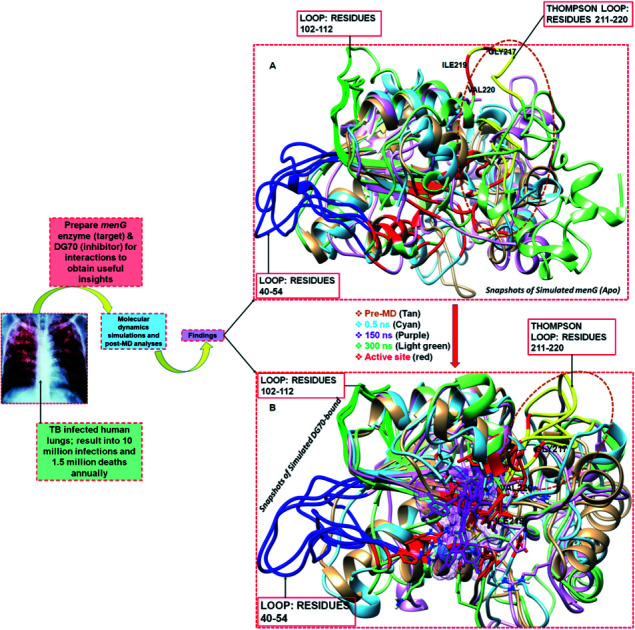

## 1 Introduction

**Fig. 1:**
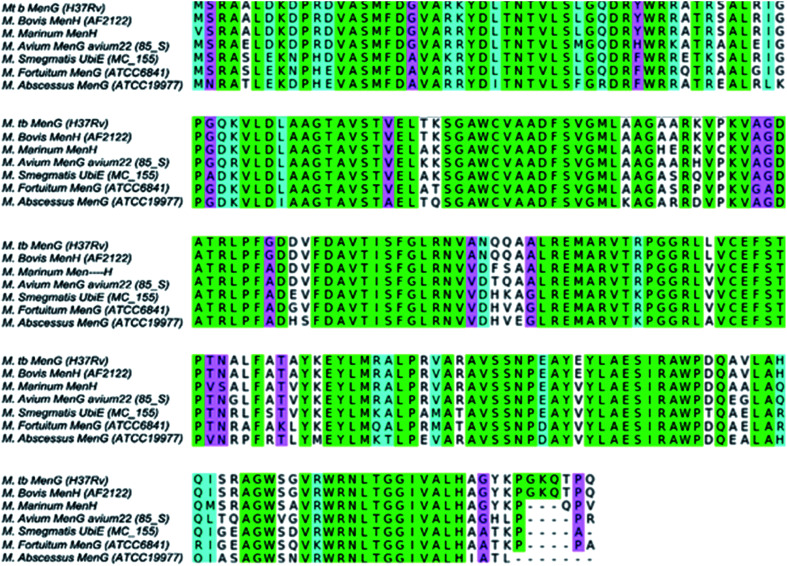
Sequence analysis of *Mycobacteria menG* showing ≥80% conserved amino acid residues.^16^

*Mycobacterium tuberculosis* (*Mtb*) is the world's deadliest infection after the renascent human immunodeficiency virus/AIDS.^1^ About two billion latent *Mtb* in man kill 2–3 million people annually.^1^ Presently, more than 484 000 new cases of multidrug and extensively resistant (active) TB cause about 1.5 million human deaths every year.^2,3^ More disturbing is the mutation-mediated and acquired drug resistance by the newer antitubercular drugs including linezolid, bedaquiline, (BDQ), *etc.*^4,5^ More than one-half of the MDR/XDR-TB drugs have failed due to mutation.^6^ In the quest for a promising novel drug over the years in this area, researchers have focused on protein–drug and protein–protein interactions for possible tuberculosis cures using various techniques and methodologies through targeting protein biocatalysis.^7,8^ While a lot of studies have reported the targeting of constituents of *Mtb* cell walls, most often, the mycolic acid synthesis, only a few reports seem to be available on the study of the inhibition of the *Mtb* respiratory pathway using experimental and computational tools such as molecular dynamics (MD). BDQ is the first respiratory inhibitor that acts by binding to the oligomeric and proteolipid subunit C of *Mtb* ATP synthase (*atpE gene*).^5,6^ BDQ was very effective against both the susceptible and multi-drug resistant TB.^9^ However, there is a high prevalence of mutations in *Mtb* genes, including *atpE*, *atpC*, *mmpR* (Rv0678) to bedaquiline. A whole-genome sequence has revealed a genetic signature of BDQ resistance in a clinical *Mtb* isolate C; a microhetero-resistance found in a targeted deep sequencing analysis.^10,11^ Similarly, a previous study shows that *Mtb* demethylmenaquinone methyltransferase, *menG* (rv0558) is a potential target and involves the biosynthesis of menaquinone (MK), the substance required for the cell maintenance of the prokaryotic including *Mtb*.^12–14^ The amino acids of *Mycobacterium tuberculosis menG* (rv0558) protein are conserved and distributed among *Mycobacterium species* homologs^15^ as shown in Fig. 1.^13^

*MenG* is a membrane domain-associated enzyme and belongs to the SAM-dependent methyltransferase (MTase) superfamily comprising of an α/β/α fold structure.^17,18^ MTase domain is an important target in drug design. *Mtb menG* encodes *S*-adenosyl-l-methionine (SAM)-dependent *menG* (Rv0558) that catalyzes the biosynthesis of menaquinone [in the form of MK9(H_2_)] required for respiration through *C*-methyl transfer from the SAM.^19^ After the methylation catalysis of the *menG* enzyme, SAM converted to *S*-adenosyl homocysteine (SAH) which leaves the protein domain.^18^ SAM-bound methyltransferases is a Class I enzyme and may possess at least two or more GXG motifs located whereby one is located in the first β-sheet and the second is bound another β-sheet.^20,21^ Puffal *et al.* (2018) also reported that *Mycobacterium smegmatis* (*Msmeg*) *menG* has 234 amino acids and GXG motif.^22^ Methyltransferases include Rossmann-like α/β (Class I), TIM α/β-α/β (Class II), tetrapyrrole methylase α/β (Class III), SPOUT α/β (Class IV), *etc.*^23^*MenG* is a Class I methyltransferase with remarkable structural consistency and a 10% primary structure similarity.^21^

DG70, a biphenyl amide, is a chemotype compound that inhibits the catalytic methylation of *Mycobacterium tuberculosis* demethylmenaquinone methyltransferase enzymes. The whole cell-based screen of a *Mycobacterium menG* using a putative *PcydAB reporter strain* and HRMS analysis identified was used to determine the potency of DG70 as a *menG* inhibitor.^24^ Moreover, this compound is therapeutically active against both the clinical and laboratory drug-susceptible and drug-resistance *Mtb strains*.^13^

In drug design and development, protein dynamics study often provides information that may result to the desired novel target and its mechanism of catalysis (substrate binding) or discovery of an Mtb enzyme inhibitor and its mode of action (MOA).^25–27^ This study investigates the changes in the conformational structure of *menG* and the inhibition of its catalytic activity with DG70 using computational approaches to understand its potentiality as a pharmacological target to eradicate tuberculosis. We created a homology model of *menG* enzyme and studied its ligand-unbound, substrate-bound (DMK9), and ligand-bound (DG70) systems over 300 ns molecular dynamics (MD) simulations at the atomistic level.

### 1.1 Concise DG70 structural chemistry: probable advantage over the frontline drugs

The study of *menG* inhibitors and specifically, the DG70, a potential drug for the treatment of TB, is relatively new. Hence, it is considered a probable research area by which this disease eradicated. Besides the mutation-mediated resistance in protein residues, including *menG* that militate the cure of TB, the inhibitor activity, which is partly determined by its structure, can also serve as a hindrance. We compared the chemical structures of DG70 and BDQ to provide a probable justification of why DG70 showed a therapeutic advantage over the BDQ drug.

**Fig. 2:**
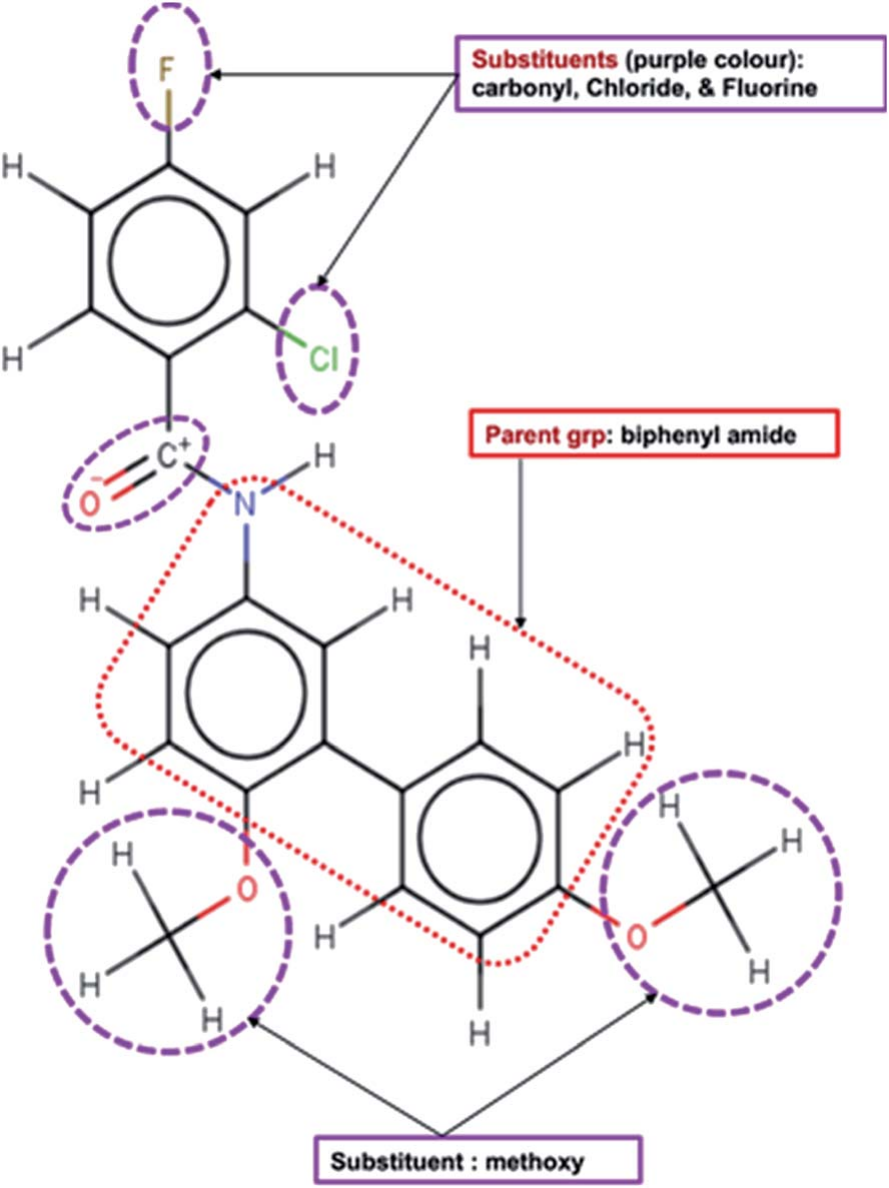
*MenG* inhibitor (DG70) and potential interacting functional groups shown in dotted lines.

The functional elements of chemical structures (Fig. 2) determine their potential therapeutic efficacies against infection or disease. They are essential factors in drug design and development. First, the groups have the potentials to form strong interactions with the active site of *menG*. For instance, high electronegative atoms of DG70 can form strong bonding interactions with the protein interacting residue thereby preventing the compound from moving in and out of the active site. Moreover, the oxygen and hydrogen atoms of the methoxy group and the phenyl rings can form strong conventional (carbon) hydrogen bonds, electrostatic, and pi–sigma interactions. There can also be a formation of alkyl and mixed/pi–hydrophobic interactions between DG70 and the interacting residues.

## 2 Computational methods

### 2.1 Homology modeling and binding pockets identification

#### 2.1.1 *Mycobacterium tuberculosis* menG homology modeling

There was no closely related structure of *menG* protein found in the protein data bank (PDB) during this study. Most protein PDB structures have less than 30% sequence identity in the homologs of the *Mtb menG* sequence.^28^ Moreover, the model reported previously by Sukheja *et al.* (2017) was also not available for use. Hence, we conducted a systematic search for possible structural templates by uploading the *menG* protein sequence (accession code: P9WFR3) obtained from the UniProt database^29^ onto the BLAST. The templates of the P9WFR3 sequence were identified from NCBI using the BLASTp (accessed on 28 June 2019).^30–32^Table 1 showed the sources, the accession codes, and the selection criteria for choosing the template. *Mtb menG* protein templates share some sequence similarities with the SAM-dependent methyltransferases crystal structures.

**Table 1:** Criteria for choosing the templates for modeling the *Mtb menG* protein^a^

Template source	PDB code	Query cover (%)	Sequence identity (%)	*E*-Value
*Saccharomyces cerevissia* sp	*4OBW.A*	91	32.51	0
*Lechevalieria aerocolonigenes*	*3BUS.A*	92	23.81	0
*Saccharomyces cerevissiae* spp	*40BW.A*	99	37.26	0
*Streptomyces luridus* spp	*3OU2.A*	38	21.99	0

a The templates also belong to the transferase family.

The MODELLER software version 9.21, an add-on in UCSF ChimeraTools-1.13.1 was used to create the homology modeling of the target protein in which all the four templates selected to build the model.^33,34^ We performed multiple sequence alignments using CLUSTALW online application,^35^ which uses a BLOSUM matrix and penalties of 10 and 0.1 for gap opening and extension, respectively. The multiple sequence provides evidence that the full length of chain A *Saccharomyces cerevisiae* spp is the best template with the highest identity score. To obtain a 2D secondary structure of *menG* enzyme, we uploaded its sequence to UCSF Chimera version 1.13.1 where structural alignment and comparison, matchmaking, match-aligning, and modeller (homology) were used to build the model. Five models named A, B, C, D, and E having zDope (kcal mol^−1^) −0.32, −0.28, −0.14, −0.06 and −0.00 respectively were generated. Model A (zDope = −0.32) was chosen since it is the best one because it has most negative value. The high structural similarity between the homolog and the template used give credence to the modeling strategy used. A Ramachandran plot for the analyses of bond angles and torsional strain generated using MolProbity.^36^ Of all residues, MolProbity results favoured 87.1% and allowed 96.1% (223/232), and nine outliers residues. The active sites were obtained using Metapocket2.0 and validated using Raptor-X, GalaxyWeb, and 3DLIGANDSITE resource. The predicted active site residues were not contained in the outliers.

#### 2.1.2 Binding pockets identification

Four online resource programs were employed to predict the active sites of *menG* protein structure. Metapocket2.0 server deployed to identify the druggable pockets^37^ (Adeniji, Olotu, & Soliman, 2018) while cross-validations of the binding sites sorted out using Raptor-X web,^39^ 3D-Ligandsite web,^40^ and GALAXYWEB server.^41^ Metapocket2.0 is a consensus method that incorporates four other methods, including the LIGSITE, PASS, Q-SITEFinder, and SURFNET to generate protein surface for clefts and cavities. Raptor-X is a web server with evolutionary information. It employs a powerful in-house deep learning model Deep Convolutional Neural Fields (DeepCNF) to predict the secondary structure and disorder regions, solvent accessibility.^42^ GALAXYWEB server predicts protein structure from a sequence by template-based modeling and refines loop or terminus regions by *ab initio* modeling. It relies on the method tested in the 9th Critical Assessment of techniques for protein structure prediction as Seok-server.^43^ GALAXYWEB prediction method generates stable core structures from multiple templates and rebuilds unreliable loops or termini by using an optimized-based refinement method.^44^

### 2.2 System preparations and docking calculations

DG70 acts against the *menG* catalysis of methylation of demethylmenaquinone (DMK9) to menaquinone (MK9) both *in vitro* and *in vivo*. The catalytic methylation of DMK9 by *Mtb menG* produces MK9 that is required for energy. Hence, we considered docking calculations were performed on ligands; substrate (DMK9) and inhibitor (DG70). DMK9 and DG70 were drawn in MarvinSketch-17.21 (http://www.chemaxon.com/) and converted to a mol2 format. DMK9 and DG70 were separately assessed in Molegro Molecular Viewer (MMV)^45,46^ to ensure that bond angles and hybridization state were corrected. The ligands minimized using the steepest descent method and GAFF force field add-on in Avogadro.^47^ The systems were subjected to molecular docking using the predicted active site to refine and reduce the probable false positives due to ligands and to assess the geometrical feasibility at the site. The pose with the lowest negative value was chosen based on the interactions and binding affinities of the ligands. Both DG70 and DMK9 docked using AutoDockTools-1.5.6 graphical user interface^48^ to defining the grid box for the active site; the spacing of 5 Å and size 122 × 106 × 86 pointing in *x*, *y*, and *z* directions. Similarly, the grid box for the same active site of DG70 docked defined as having a center (−37.66 × −45.85 × 37.45) and dimensions (120 × 106 × 72) the spacing of 5 Å and size 124 × 106 × 72 and pointing in *x*, *y*, and *z* directions respectively. Docking calculations using the Lamarckian genetic algorithm carried out with AutoDock Vina.^48^ Using UCSF ChimeraTools-1.13.1, atom types were assigned, Gasteiger charges added to the ligands, and merged non-polar hydrogen to carbon atoms. The water molecules were removed from the protein and added polar hydrogen and the ligands docked into the active site of *menG*. Finally, docking was validated based on the lowest energy pose.

### 2.3 Molecular dynamics (MD) simulations

Molecular dynamics simulations find applications in the study of the atomistic motions of biological systems and furnish us with the understanding of the physical movements of atoms and molecules. MD provides the interpretations of molecular processes within the biosystems.^49,50^ We performed the MD simulations using the GPU version of the PMEMD.CUDA engine provided with the AMBER package, FF18SB variant of the AMBER force field used to describe the protein.^51^ In applying the restrained electrostatic potential (RESP) and the GENERAL AMBER Force Field (GAFF) procedures, the partial charges were added to the ligands using ANTECHAMBER.^18^ The LEAP module in AMBER 18 was used to neutralize and solvate the free *menG* and ligand-bound systems by adding hydrogen atoms [H^+^], sodium ions [Na^+^], and chloride [Cl^−^] counter ions.

Furthermore, all atoms explicit solvation was carried out in an orthorhombic TIP3P box of water molecules size 10 Å. The procedure considered initial minimization of 2500 steps with an applied restrained potential of 500 kcal mol^−1^. Additional 5000 steps of full minimization were carried out by conjugate algorithm without restraining conditions. The systems were gradually heated, starting from 0 to 300 K for 50 ps to maintain a fixed number of atoms and volume considering a canonical ensemble (NVT). Using 1 bar pressure provided by the Barendsen-barostat, the SHAKE algorithm utilized to constrict the hydrogen bond constraint.^52,53^ The total time for the MD simulation was 300 ns with a time step of 2 fs using constant pressure of 1 bar, a 300 K, and Langevin thermostat. The coordinates Apo-*menG* and bound-*menG* complexes were each saved every 1 ps, and the trajectories were analyzed using the CPTRAJ and PTRAJ module in AMBER 18/GPU. The trajectories analyzed for RMSD, RMSF, DSSP, principal component and analysis, and radius of gyration using CPPTRAJ and PTRAJ. The structural and visual analysis^54^ done by employing the graphical user interface of UCSF Chimera and Discovery Studio 2019 Client to analyze the binding mechanism of the ligand-bound systems,^55^ while the data plotted with MicroCal Origin 6.0 data analysis software.^53,56^

### 2.4 Post-dynamics analysis

#### 2.4.1 Binding free energy calculations

Molecular dynamics employs the differences in the free energy to study the mechanism of biological processes.^57^ The binding free energy (BFE) method is important in the computational study of protein dynamics to explore their binding mechanism with ligands thermodynamically.^58^ The BFE of the bound system calculated using the molecular mechanics/GB surface area method (MM/GBSA) to estimate their binding affinities.^51^ The energies considered over 300 000 snapshots from the 300 ns trajectories. This free binding energy (Δ*G*) calculated for DMK9- and DG70-bounds *menG* and expressed thus:Δ*G*_bind_ = Δ*G*_complex_ − Δ*G*_receptor_ − Δ*G*_ligand_Δ*G*_bind_ = *E*_gas_ + *G*_sol_ − *T*Δ*S**E*_gas_ = *E*_int_ + *E*_vdw_ + *E*_ele_*G*_sol_ + *G*_GB_ + *G*_SA_*G*_SA_ + γSASA*E*_gas_ denotes the gas-phase energy-internal energy: Coulomb (*E*_ele_) and van der Waals energies (*E*_vdW_). The *E*_gas_ estimated from the FF18SB force field terms. Polar and non-polar states' energy contributions accounted for the solvation free energy, *G*_sol_. The non-polar solvation energy, SA. *G*_SA_ was determined from the solvent-accessible surface area (SASA), using a water probe radius of 1.4 Å. In contrast, the polar solvation, *G*_GB_, was obtained by solving the GB expression. *S* denotes the total entropy and *T*, the temperature of the systems. The contribution of each residue to the total binding free energy obtained at the predicted active site by carrying out per-residue energy decomposition at the atomic level using MM/GBSA method in AMBER 18.^59^

#### 2.4.2 Receptor–ligand interactions systems

Analysis of *menG* active amino acids interaction network with DMK9 and DG70 carried out using the receptor–ligand interaction add-on available on the Discovery Studio Visualizer 2019 Client.^53^ The interactions depicted using the snapshots taken at different periods of 300 ns MD trajectories for DMK9- and DG70-bound systems and subsequently visualized using BIOVIA Discovery Studio software to obtain the molecular forces between the atoms of the ligand and interacting residues. These interactions may provide important information that would help to discover if *menG* is a potential target and contribute to the overall design and development of contributing to the development of drugs. Using this software allows us to depict the interacting residues and the interaction types within the bound systems.

#### 2.4.3 Conformational fluctuations of menG systems

The RMSF is the fluctuations of individual protein residues to their average positions within a given molecular dynamic simulation.^60^ It provides an understanding of the flexibility of various regions of *menG* while binding to the ligands. Mathematically, RMSF is as follows:
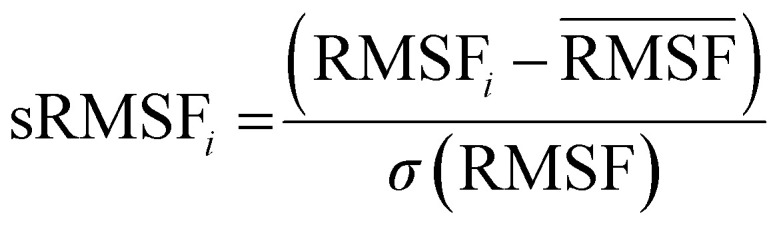
RMSF_*i*_ is the RMSF of the *i*^th^ residue from which the RMSF of the average is taken and divided by the RMSF's standard deviation [*σ*(RMSF)] to give resultant standardized RMSF [sRMSF_*i*_].

#### 2.4.4 Radius of gyration (RoG)

The radius of gyration of both the Apo and the bound system were determined to further examine the stability of the system through a compactness test. Statistically, the average radius of gyration (RoG) predicts the dimensions of biomolecule as it reflects the molecular compactness of a system to its shape by describing the RMSD of the atoms from the common center of gravity of a given protein molecule.^61^ The compactness of Apo and bound systems assessed along 300 ns MD trajectories by taking the average over 300 000 frames. The equation below expresses the estimation details of RoG is estimated:
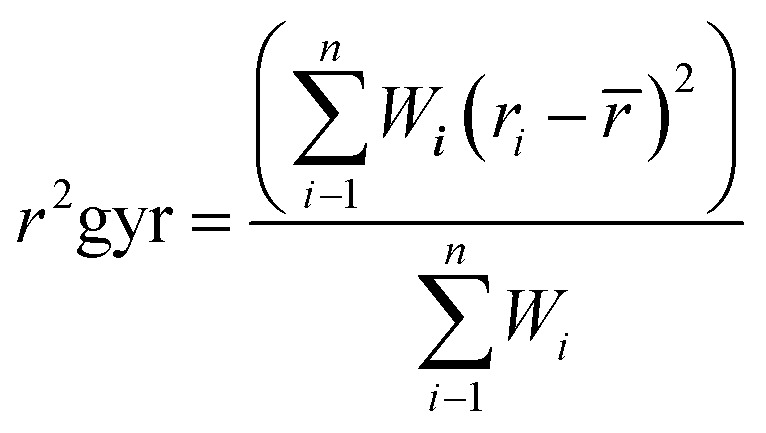


The terms in the equations include the position of the *i*^th^ atom (*r*_*i*_), mass/weight of each atom (*W*), and the center mass of atom *i* (*r*). The mean value calculated by taking the RoG values over the number of frames in each trajectory.

#### 2.4.5 Principal component analysis (PCA)

The principal component analysis is an important post-MD analysis that describes the atomic displacement and the loop dynamics of a 3D protein. It represents the magnitude (eigenvalues) and direction (eigenvectors) of the motion of protein.^62^ Before processing the *menG* PCA, the MD trajectories of free enzyme, DMK9-, and DG70-bound complex enzymes were stripped of solvent and ions using an integrated PTRAJ module in AMBER 18. Considering the dynamics of the C-α atom of *menG*, the first two principal components (PC1 and PC2) were computed and the conformational patterns of the free and bound *menG* systems projected along the first two eigenvectors, *i.e.* ev1/PC1 *vs.* ev2/PC2 using the C-α atoms Cartesian coordinates.^53^

## 3 Results and discussion

### 3.1 The homology structure of *Mtb menG*

The preparation of the 3D structure of a protein is the first step in rational drug design.^63,64^ However, there was no available crystal structure of menG for this study. Moreover, a previously reported homology model of *Mycobacterium tuberculosis* menG13 was also not available. Hence, a homology model was built using 4 templates; crystal structures of two 4OBW, 3BUS, and 3OU2 obtained at resolution range between 1.5–2.4 Å from the protein data bank (PDB). The criteria (Table 1) for choosing the quality templates^64^ produce successful menG protein models from which a model with the best zDope (−0.32 kcal mol^−1^) was chosen to further this study.

The *menG* (rv0558) sequence consists of 234 amino acids corresponding to the *saccharomyces cerevisiae* transferase amino acid residues (74–301). The structural features of a protein are a good indicator of its functions. *Mtb menG* showed two conserved cysteine residues (CYS76 and CYS146) that tend to form a disulphide bridge, thus act as activators in the protein. Site-directed mutagenesis determination and ligand affinity analysis of *menG* could reveal a probable formation of a cysteine bridge. Furthermore, the *MenG* sequence is characterized by hydrophobic amino acids including especially at the active residues.

**Fig. 3:**
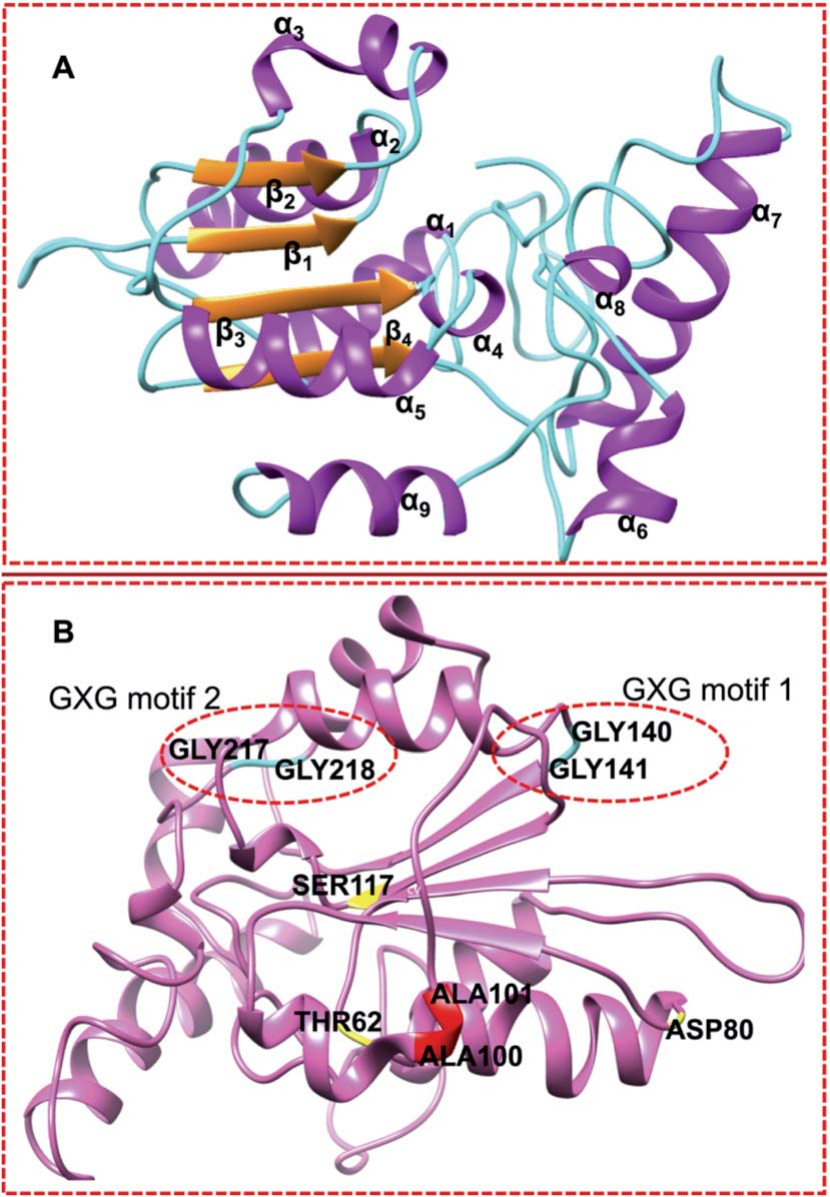
(A) Secondary structural features of *Mtb menG* showing 4 parallel β-strands (orange) located at the center and surrounded by 9 α-helices (purple). (B) GXG motifs residues: GLY140, GLY141, GLY217 (interacting residue), and GLY218 (cyan); UniProt annotated residues of the co-factor binding residues (red) ALA100 and ALA101; and the substrate-binding residues (yellow) THR62, ASP80, and SER117 among the active site residues.

Topologically, the superimposition of homology *menG* structure and *4OBW* template showed high similarity. The secondary structures of a protein including *menG* are a function of its different conformations. The structural features of protein provide a means of understanding the molecular interactions.^20^ In this study, we observed that the *Mtb menG* possesses parallel β-strands and fully formed α-helices (Fig. 3A); four beta-strands (4_β_) that are surrounded by nine-alpha (9_α_) helices. This finding correlates with Class I methyltransferase.

Fig. 3B showed two GXG motifs located on two different loops of *Mtb menG* as against the β-sheet that was previously reported. These motifs are conserved in the homologs of *Mycobacterium tuberculosis menG*. Moreover, motif GLY217 played a binding role at the active site of this protein.

### 3.2 Active site determination and validation

**Fig. 4:**
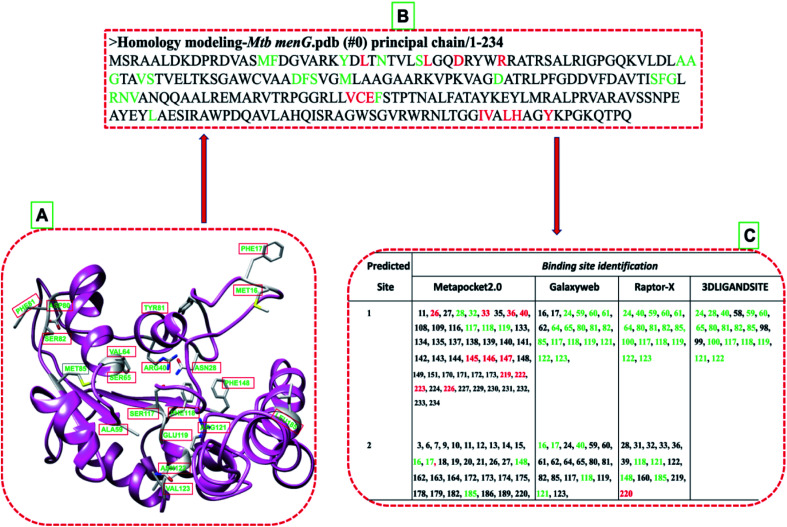
*MenG* predicted active sites (green) and the interacting residues (red). (A) Secondary structure illustration *menG* of the active site (common residues by predictors). (B) The primary structure displays predicted active residues (green) and the interacting amino acids (red). (C) provides the corresponding numbers of the predicted active amino acids.

The ligand-binding sites of the model with the highest zDope score (−0.32) predicted to identify the corresponding pocket sites using Metapocket2.0, raptor-X, 3D-LIGANDSITE, and GALAXY (Fig. 4C). The negative zDope score of −0.32 obtained upon binding the *menG* inhibitor indicates a reasonable accurate structure (native-like structure) and implies that the complex is very stable and reliable such that the DG70 is bound to the protein without moving in and out of the active site. The selected residues (Fig. 4A) were considered the active site and used for the molecular docking and modeling of the interaction of *menG* residues with the ligands. The active site determined for the substrate and the *menG* inhibitor. The site validated by generating a Ramachandran plot (ESI Fig. S2†) of the active site, which showed that they did not fall within the outlier region.

### 3.3 Molecular docking of DMK9 and DG70

The Uniprot annotated THR62, ASP80, and SER117 to be among the substrate active site residues and ASP100 and ALA101 to be among the co-factor active site residues.^44^Fig. 4C showed that the predicted active site residues agreed with UniProt annotation, though it did not differentiate which residues will bind to the substrate or co-factor. Hence, we employed a previously reported criterium for choosing the active site residues.^38^ Moreover, our predicted active site is similar to the one (residues: ARG3, ASP7, VAL20, LYS23, ARG40, ARG48, ALA60, TRP75, GLY99, PHE118, ARG121, VAL136, ALA181, SER188, and ARG190) that was previously reported by Sukheja *et al.*, (2017), having residues ARG40, ALA60, PHE118, and ARG121 in common.

Molecular docking is a major computational tool that predicts the multiple orientations of a ligand at a binding site of receptors.^65–67^ The substrate (DMK9) and DG70 (inhibitor) docked separately in the active pockets of the *menG* and the pose that gave the most favorable conformations (highest negative value) considered for the molecular simulation.

The molecular docking of DMK9 and DG70 on the active pocket of *menG* showed multiple orientations within the binding sites. Although the interacting residues used in this work were like those residues reported the Sukheja group, our Ramachandran plot showed that ASP7, ASP9, and PRO10 were outliers. Whereas, the outliers reported as interacting residues. Rather than using the binding site predicted in the previous work, we chose to predict a new active site of menG protein and validated it before use. DG70 docked on the most favourable binding mode (score = −7.1 kcal mol^−1^), and DMK9 produced a score of −5.5 kcal mol^−1^ using the same active site. We observed that both ligands interacted with different residues suggesting that one ligand docked at the site of the co-factor site and the second one docked at the substrate's site.

### 3.4 Per-residue energy decomposition (PRED) and ligand interactions with *menG*

**Fig. 5:**
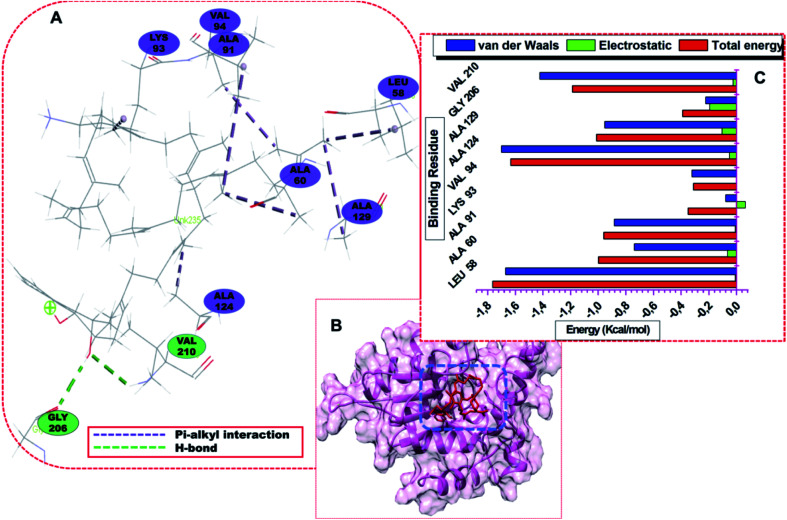
(A) Visual representation of the DMK-*menG* interaction network (colour online) and (B) structural view of DMK that bound to the *menG* the active site. (C) Energy contributions of the interacting residues of *menG* with DMK9.

The per-residue energy decomposition revealed the interactions between the interacting residues and the ligands.^18^ Considering the 300 ns MD trajectories, the per-residue decomposition of *menG* residues revealed various contributions of the binding site residues to DMK9 and DG70. The ligands adopted favourable orientations or morphology such that functional groups including chlorine, fluorine, –OCH_3_, –(C_6_H_5_)_2_NH–, and residue groups such as –NH_2_, –COOH, cysteine sulphur atom interacted with the active site residues formed strong bonds. Explicitly, it observes that the methyl C-atoms of side chains of LEU58, ALA60, ALA91, LYS93, and VAL94 developed stable and strong alky hydrophobic interactions with the methyl C-atom of the prenyl group of DMK9 (Fig. 5A). Also, there was the formation of a conventional hydrogen bond; the primary chain O atom of GLY206 formed hydrogen (H-bond) with H-2 of DMK9 phenyl group and the oxygen (O-2) of DMK9 created H-bond with the main chain H-bond of VAL210.

The hydrophobic residues that pocket DMK9 predominantly include Ala60, Val91, Lys93, Val94, Ala124, Ala129, GLY206, VAL210, and basic LEU58. Except for GLY206 and VAL210, the interacting residues formed alkyl hydrophobic interactions with the H-atoms of the prenyl group of DMK9. Both GLY206 and VAL210 formed conventional H-bonds (classical); backbone O atom of GLY206 bonded with the one prenyl H-atom while backbone H-atom of VAL210 bonded with the phenyl hydroxyl O atom of DMK9.

**Fig. 6:**
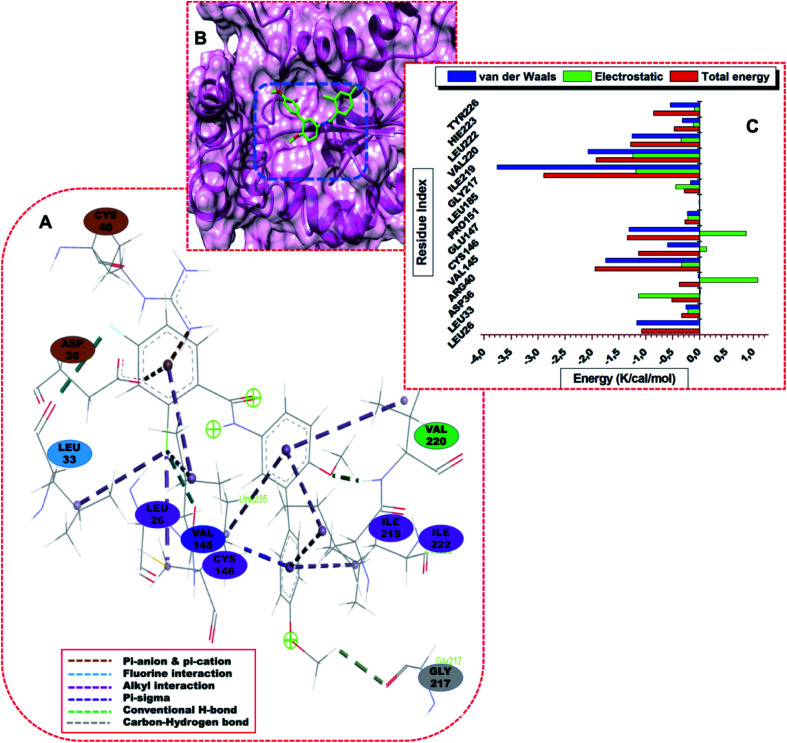
(A) Visual representation of the DG70-*menG* interaction network, (B) structural view of DG70 bound to *menG* active site , and (C) **e**nergy contributions of the interacting residues of *menG* with DG70.

The residues that pocket DG70 include TYR24, ARG40, GLN126, CYS146, and GLU147. LEU26, LEU33, VAL145, and CYS146 formed strong and stable bonds with highly electronegative chlorine (Cl) atom of DG70. Fig. 6A represents the interaction networks of the active site residues with DG70. The systems are characterized by stable hydrophobic, single halogen and hydrogen bonds. DG70 bound to the active residues including LEU26, ILE219, LEU222, and GLN235.

The resonating phenyl rings (C13–C18 and C19–C24) of DG70 formed strong pi-alkyl bonds. ASP36 and ARG40 formed pi-anion and pi-cation respectively with the phenyl (C1–C6) ring of DG70. Moreover, LEU26 and VAL145 formed alkyl hydrophobic and pi–sigma interactions with chlorine (Cl-7) and phenyl ring, respectively. Additional interaction is stronger interaction between residue LEU33 and fluorine (F-8) of DG70. Other significant interactions are the formation of conventional and non-classical H-bonds that were formed from the backbones of VAL220 and GLY217 with methyl hydrogen (H-44) and methoxy oxygen (O-27) atoms of DG70, respectively.

Using the MM/GBSA approach, we obtained individual residue-based contributions from the decomposition of the total binding protein energy (BPE) for DG70. We estimated the van der Waals (vdW) and electrostatic (elec.) interactions. The two forces reveal the residue and energy that produced an overall greater impact on the total binding energy. The per-residue decomposition of *menG*-DMK9 and *menG*-DG70 depicted as seen in Fig. 5C and 6C, respectively. The binding strength and the extent of stability of DG70 at the pocket site depend on the intermolecular forces that occurred between the inhibitor and the active site residues. LEU58 (−1.68 kcal mol^−1^), ALA60 (−1.0 kcal mol^−1^), ALA91(−0.8 kcal mol^−1^), ALA124 (−1.58 kcal mol^−1^), ALA129 (−1.05 kcal mol^−1^), and VAL210 (−1.25 kcal mol^−1^) contributed higher energies to the complex, while VAL94 (−0.35 kcal mol^−1^) and GLY206 (−0.4 kcal mol^−1^) contributed less energy. Therefore, the van der Waals interactions due to residues 58, 60, 91, 124, and 210 contributed to the high energy interaction of the covalent system (Δ*G*_bind_ = −31.64 kcal mol^−1^).

The per-residue interaction energy of the *menG*-DG70 system and the residues that contributed higher energies towards the complex include LEU26 (−1.15 kcal mol^−1^), VAL145 (−1.9 kcal mol^−1^), CYS146 (−1.2 kcal mol^−1^), GLU147 (−1.35 kcal mol^−1^), ILE219 (−2.8 kcal mol^−1^), VAL220 (−1.8 kcal mol^−1^), LEU222 (−1.8 kcal mol^−1^), HIS223 (−0.5 kcal mol^−1^), and TYR226 (−0.65 kcal mol^−1^). Although the per-residue decomposition did not show the formation of a disulphide linkage between CYS146 and CYS76, there was a strong alkyl hydrophobic interaction between carbon–sulphur (C–S bond) of CYS and Cl atom of DG70 (Fig. 6A). In contrast, residues LEU33 (−0.35 kcal mol^−1^), ARG40 (−0.4 kcal mol^−1^), PRO151 (−0.25 kcal mol^−1^), and GLY217 (−0.28 kcal mol^−1^) contributed less energies while LEU185 did not influence the complex energetically. Therefore, the vdW interactions due to residues 145, 146, 147, 219, 220, 222, and 226 and the elec. interactions due to ASP36 (−1.15 kcal mol^−1^) contributed to the huge stable covalent system (Δ*G*_bind_ = −32.48 kcal mol^−1^). The Δ*G*_bind_ for the *menG*-inhibitor system showed that DG70 is highly stable. This stable and strong interactions between DG70 and *menG* have a high impact on its inhibitory efficacy. Hence, we can correlate this results with the previous experimental report; the minimum inhibitory concentrations (MIC) of DG70 against drug-susceptible *Mtb* H37Rv (be 4.8 μg mL^−1^) and drug-resistant *Mtb* strains (1.2–9.6 μg mL^−1^) respectively.^16^ These strong interactions and high stability of *menG*-DG70 prevent the synthesis of MK9. However, we improve the binding affinity predictions of the complexes by subjecting the systems to 300 ns MD simulations to have more realistic flexible *menG* in an implicit solvent. Protein–ligand systems were analyzed using the accurate MMGBSA/free binding calculations to obtain the most favourable pose of DMK9 and DG70 at the *menG* active sites. It observes that DMK9 and DG70 interacted with their respective *menG* active sites throughout the molecular simulations.

### 3.5 Free energy calculations of *menG*-DG70 binding affinity

The binding free energy provides various distinct energy contributions within the binding pockets and the binding orientations that gave the best intermolecular interactions at protein active sites. The total binding free energy for bound systems; *Apo*DMK9 and ApoDG70 systems obtained using the MM/GBSA approach. We further determine the interactions networks of the DG70-*menG* (denoted ApoDG70Exp) reported by Sukheja *et al.* and depicted its residue–ligand interactions to compare with our results.

The docking pose score (kcal mol^−1^) for *Apo*-DMK9, *Apo*DG70, and ApoDG70Exp- were −8.1, −7.1, and −5.5, and respectively. The thermodynamic energy contribution of DMK9 and DG70 to their respective complex and the total binding free energy determines their stability at *menG* active site. Table 2 summarizes the binding free energies of the complexes contributed by the protein and the ligand.

**Table 2:** Thermodynamics analysis of m*enG*-DMK9 and *menG*-DG70 interactions^a^

Complex	Energy components (kcal mol^−1^)				
	Δ*E*_vdw_	Δ*E*_ele_	Δ*G*_gas_	*G*_sol_	Δ*G*_bind_
ApoDMK9	−16.87	−13.92	−15.61	−30.69	−31.64
ApoDG70	−16.09	−13.91	−15.52	−31.50	−32.48
ApoDG70Exp	−16.13	−13.99	−15.60	−30.45	−31.41

a ApoDG70Exp is the same as *menG*-DG70 system reported by Sukheja *et al.* (2017) whose thermodynamics data was obtained to compare with that of ApoDG70 (inhibitor-bound system) studied in this work. ApoDMK9 is the substrate-bound system.

We docked DG70 on the active site, ran 300 ns MD simulations, and analyzed the PRED of ApoDG70Exp complex. Fig. S2 (ESI†) showed the residues–DG70 interactions and their energy contributions. The residues that contributed high energies towards the complex include THR62 (−0.08 kcal mol^−1^), ASP80 (−0.12 kcal mol^−1^), SER117 (−0.05 kcal mol^−1^), ASN122 (−0.13 kcal mol^−1^); PHE118 (−0.02 kcal mol^−1^) and ARG121 (1.10 kcal mol^−1^) contributed less energies while VAL20, ALA181, and SER188 did not influence the complex energetically. The elec. interactions due to residues 20, 80, 117, and 112 and vdW interactions due to residues contributed to the stable system (Δ*G*_bind_ = −31.41 kcal mol^−1^), which the *menG*-inhibitor system is stable. For this active site, interactions were mainly electrostatics in comparison to our study where the vdW interactions contributed more to the binding of DG70.

Thermodynamically, the analysis of the binding free energy of the DG70-*menG* systems showed proximity with the binding free energy obtained (Table 2). Taken together, we can infer that our predicted active site is a potential site to consider for the inhibition of the catalytic activity of *menG* protein by DG70. The most favourable binding free energies (kcal mol^−1^) were; −32.45, −31.41, and −31.64 and these data thus agreed with molecular docking scores.

### 3.6 Conformational stability of *menG* apo and bound systems

MD simulations provide useful information about the structures and dynamics of biomolecules that may contribute to the development of a drug in design and the overall achievement of the cure of TB. Specifically, proteins undergo many biological processes to regulate their internal components and maintain cellular functions. For the three studied systems, the conformational changes due to the atomistic deviations estimated using C-α backbone root mean square deviation. This system explains the structural stability and system convergence of the biological systems. For the bound or unbound proteins, attaining the structural stability requires longer timescale MD simulations. The C-α atom RMSD metric used to estimate the stability of unbound *menG* (Apo), DMK9-bound (ApoDMK9), and DG70-bound (ApoDG70) systems.

**Fig. 7:**
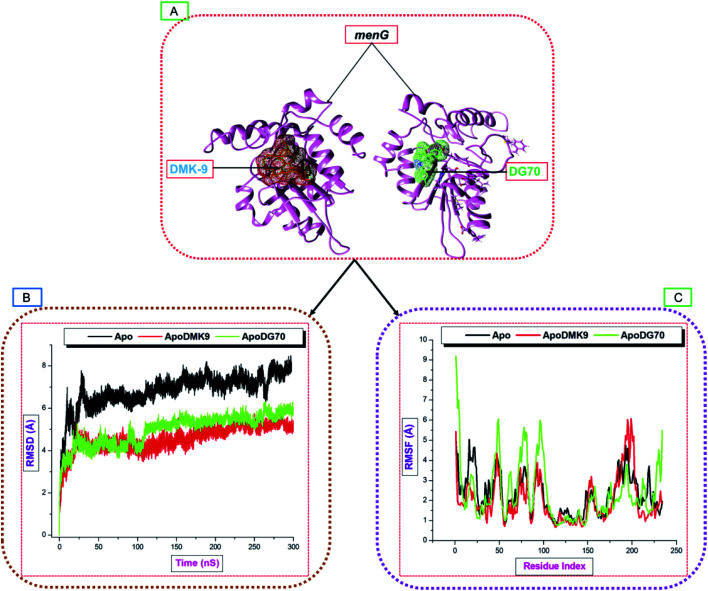
(A) DMK (red) and DG70 (green) bound to menG active site. C-α backbone RMSD (B) and RMSF (C) plots of Apo and the complexed systems ran for 300 ns MD simulations.

The RMSDs of the alpha carbon (C-α) backbone of both Apo and bound systems determined throughout 300 ns MD simulations. Fig. 7B is a graphical representation of C-α atoms RMSD of the systems. The C-α RMSD backbone atoms of all the arrangements were relatively stable. However, the RMSD of the Apo (≈8.0 Å) was quite higher than the RMSD of the bound systems which converged (RMSD = ≈4.5 Å) from 0 ns up to 110 ns before the RMSD of ApoDG70 rose to 4.7 Å. The average RMSDs (Å) of Apo, ApoDMK9, and ApoDG70 systems were 6.83, 4.66, and 4.97 respectively, and the order of increasing stabilities follows Apo < ApoDG70 < ApoDMK9. The high stability of the bound systems compared with the Apo system could be because of inward pulling interactions of the residues with the bound DG70. This difference leads to structural activation and conformational transformations.

### 3.7 Conformational fluctuation of *Mtb menG* protein

The RMSF analysis furnishes us with the significance of molecular dynamics at the atomistic level and a better understanding of the conformational changes that occur upon ligand binding. The average RMSF trajectories of MD simulations demonstrate the differences in the flexibilities of the residues with or without a ligand. The RMSF of the C-α backbone for both Apo and bound systems monitored throughout 300 ns simulations. The C-α backbone atoms RMSF of each residue in Apo, ApoDMK9, and ApoDG70 calculated and represented graphically (Fig. 7C).

The average RMSF (Å) values of Apo, ApoDMK9, and ApoDG70 systems are 2.30, 2.00, and 2.40, respectively. The average RMSF of the C-α backbone in ApoDMK9 was lower than the RMSF of the Apo and ApoDG70. The fluctuations in the ApoDMK9 system occurred between residues 180–205, which correspond to the ALA91, LYS93, and VAL94 interacting regions. Similarly, distinct high fluctuations occurred between residues 1–120, which contain residues 26, 33, 36 and 40 that formed hydrogen bond, hydrophobic, and salt bridge interactions with DG70 at the active site. Moreover, the longest loop (residue 40–54) found within this region. The flexibility greatly extended in comparison with Apo, and thus confirms the loop flexibility upon DG70-binding. Another fluctuation occurred between 225–234, which may also suggest the binding effect of DG70.

### 3.8 Distribution of atoms (the radius of gyration) around the *menG* backbones

**Fig. 8:**
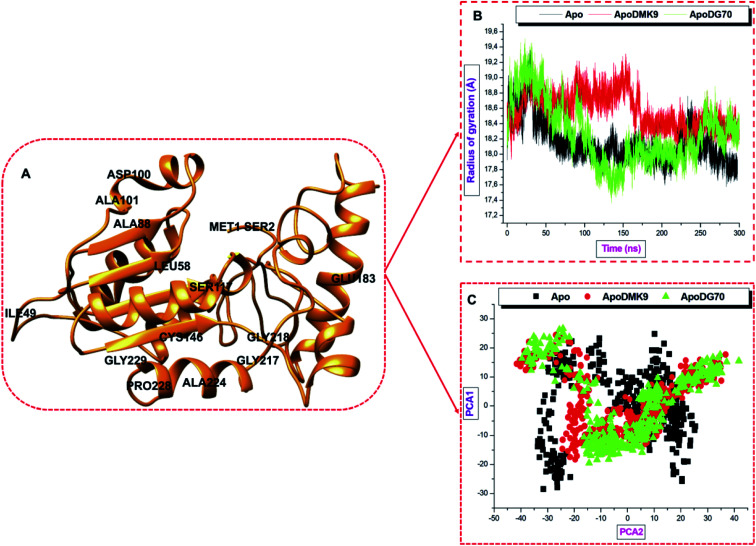
(A) Illustrates whole *Mtb menG* structure showing some residues. (B) Radii of gyrations plot of Apo and ligands-bound systems measured over a 300 ns simulation showed the differences arising in radius deviation. (C) Projection of eigenvalues of the C-α backbone, during 300 ns simulation, for Apo, DMK9- and DG70-bound conformations of menG protein along the PC1 and PC2 principal components.

The shape and folding of the Apo and bound-protein systems could further explore by measuring the radius of distribution/gyration (RoG) around the residue C-α atoms from the center of mass. Hence, this analysis provides the compactness of a protein that undergoes some dynamic forces. High RoG values depict a less tight structure and increased mobility. The radius of gyration of C-α atoms of *menG* measured before and after binding to DMK9 and DG70 in separate Apo system over 300 ns simulations. The average RoG (Å) of Apo, ApoDMK9, and ApoDG70 systems were 18.14, 18.50, and 18.27 respectively, hence implies that all the three systems showed very similar compactness. However, Fig. 8B showed the atomic distributions in ApoDG70 from 115–150 ns (low RoG, high stability) and 255–280 ns (high RoG, low stability). These correlate with the high RMSD observed between 110–160 ns and 260–300 ns.

### 3.9 Principal component analysis (PCA) of structural dynamics and motion

PCA is an essential post-dynamics analysis used to measure the conformational transitions and variations of the free (Apo) and ligand-bound protein complexes. It has applied extensively, to study the experimental data and the trajectory of the changes of simulated proteins over a time range. Moreover, the PCA tool is a robust quantitative technique that uses the covariance matrix obtained from the averaged structure using Cartesian coordinates to get the convergence of MD simulation in the space. It provides the means of determining the modes of motion and compares the positional fluctuation in the dynamically simulated structures.^62^ Lastly, PCA demonstrated the atoms' displacement and the loop dynamics of a protein and was performed on the *menG* C-α atoms using the CPPTRAJ in AMBER18 GPU to compute the first two components; PC1 and PC2 that are shown graphically in Fig. 8C. The PC1 (*X*-axis) and PC2 (*Y*-axis) represent a covariance matrix after the elimination of eigenvectors. During the simulation, each point between the single-directional motions is a unique orientation due to the overlapping of similar structural conformations.

The eigenvectors computed from the MD trajectories of the Apo and the bound systems varied greatly. The Apo system showed extensive, restricted structural motions of C-α atoms while both DMK9- and DG70-bound have broad spatial coverage which consequently showed that the free system is very rigid.^26^ Once again, this result corroborates with the stability of the systems showing the distribution pattern of the around the mass and the deviations of the system stability for the substrate- and inhibitor-bound systems. This result further demonstrates the structural loop flexibility while DG70 bound to the active site of the *menG* enzyme.

### 3.10 Loop dynamics of *menG* protein and distance metrics

The evolution of enzymes often involves sequence changes in loop regions. The study of loops can provide useful scientific significant reports in determining the protein functions, including shape, dynamics, binding properties, and physicochemical properties of proteins. They are located on the surface of the protein and often, follow a regular pattern. They are instrumental in protein study whereby loops function as connecting ends for secondary structure (α-helices and β-sheet), protein–ligand interactions, protein–protein interactions, enzyme catalysis, and recognition of sites.^68^ The biological function of loops depends on the determinants of their plasticity and the time scale of motions.

**Fig. 9:**
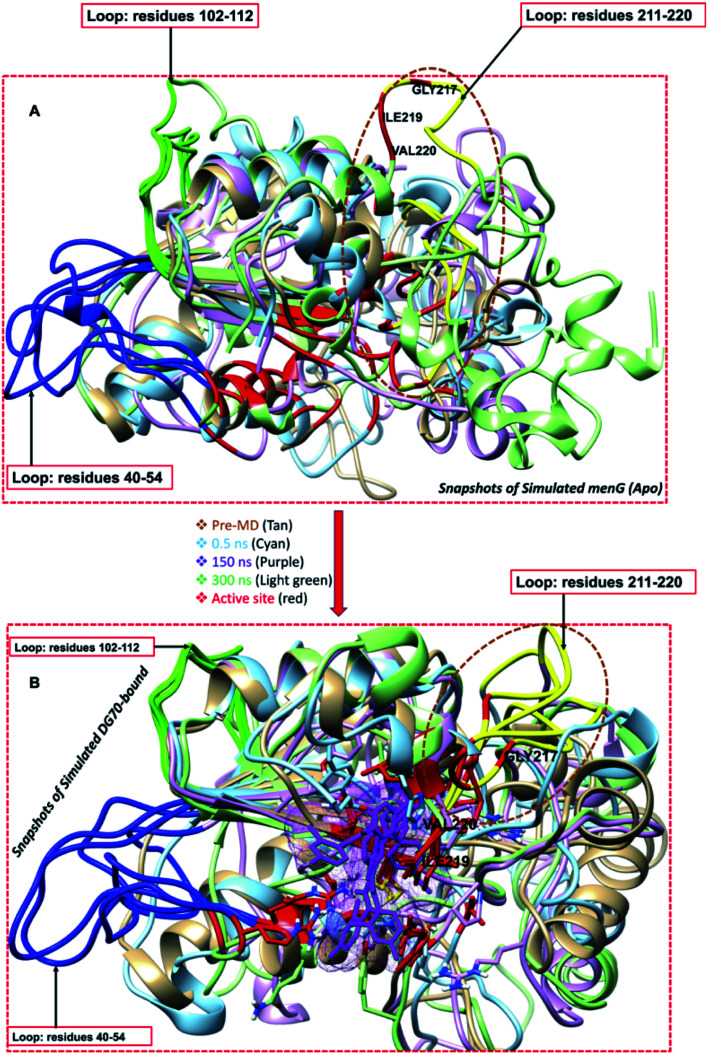
Illustration of the opening and closing conformational change of the unbound and bound *menG* protein. (A) Unbound systems superimposed snapshot at 0, 0.5, 150, 300 ns showing the active site opening of Thompson loop (yellow) and (B) bound systems superimposed picture at 0, 0.5, 150, 300 ns showing the active site closing conformation by Thompson loop (yellow).

There seemed to be little bioinformatics and the experimental findings of methyltransferase protein superfamily, especially the loops of the *menG* enzymes. Generally, the loops are not readily observable. However, we found three loops around the active site and studied them to determine their probable roles in the *menG* conformation and the binding properties. The three *menG* loops contain residues 40–54 (blue), 102–112 (green), and 211–220 (yellow) as shown in Fig. 9. The residues 40–54, 102–112 and 211–220 visually looked omega-like loop (Ω loop) and showed that the loops involved in the *menG* molecular recognition and regulatory functions.^68^ Essentially, residue 211–220 may be the common K-loop in the methyltransferase family that changes conformation upon activation to block the *S*-adenosyl-l-methionine-binding. Hence, we presumed that *menG* inhibitor bound to the active site of co-factor (SAM) against the substrate's site. Subsequently, residues 211–220, the Ω loop is referred to as the Thompson loop and used interchangeably. To determine the potential roles of these loops, we studied the visual static loop structures to observe possible conformational changes in both the Apo and inhibitor-bound systems. Fig. 9A and B showed significant conformational changes in the three loops of the Apo and the DG70-bound structures, respectively, as the simulation time increases (Pre-MD (0), 0.5, 150, and 300 ns).

Thompson loop is essential in the binding of DG70 because it contains at least three most contributing interacting residues, including GLY217, ILE219, and VAL220 of the active site and loop. The active site residues GLY217, ILE219, and VAL220 form the part of the Thompson loop that cause open conformation in the Apo system as simulation time increases. And, the residues GLY217, ILE219, and VAL220 of the Thompson loop cause closing conformational changes in the DG70-bound complex as the simulations time increases.

Therefore, the close conformation of the *menG* loop (Thompson loop) enhances the binding of DG70 and subsequently, its therapeutic effectiveness. Specifically, the changes in the formation are due to the loop residues ARG40, GLY217, ILE219, and VAL220 which control the opening and closing of the active site.

**Fig. 10:**
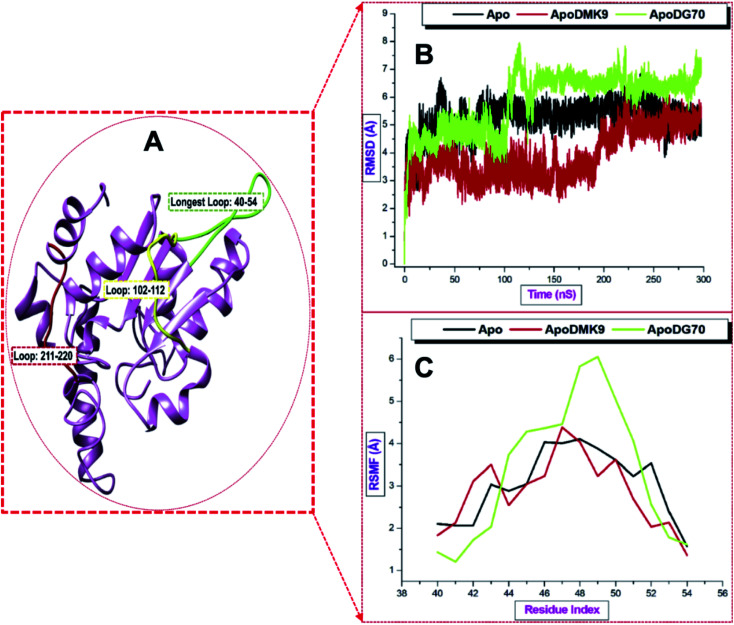
(A) Illustrates three loops in *menG* protein: residues 40–54 (green), 102–112 (yellow), and Thompson loop (red) along the trajectory. RMSD (B) and RMSF values (C) of the loops.

Second, the RMSD and RMSF were estimated to explore the dynamics of the stability and flexibility of the enzyme loops, respectively. According to the Fig. 10B, 11A and C plots, the unbound systems of the loops maintained a stable structure throughout the simulation time (RMSD < 6.5 Å) while the binding of DMK9 and DG70 induced conspicuous deviations in *menG*. The fluctuations occurred between residues 115–223, which contained GLY217, ILE219, and VAL220 that form hydrophobic and hydrogen bond interactions.

**Fig. 11:**
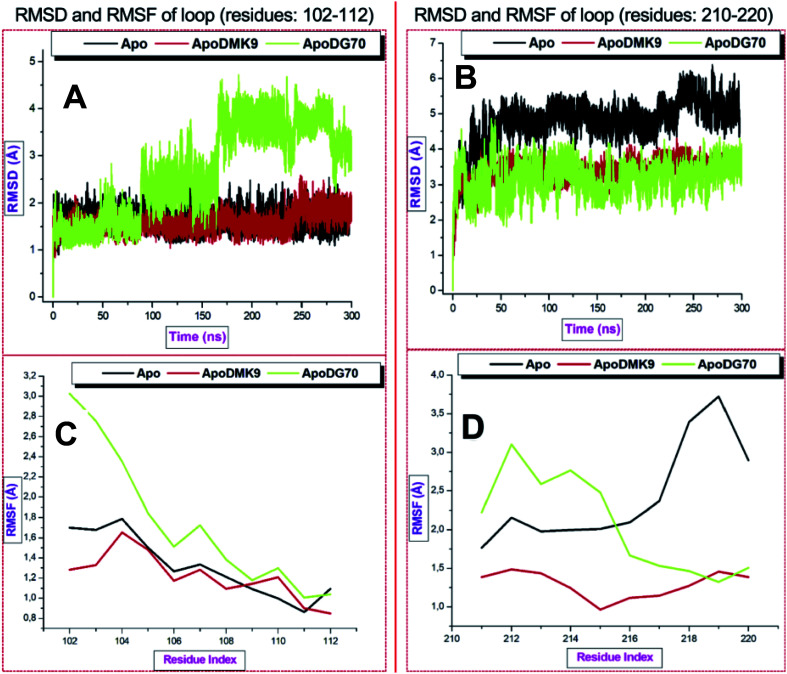
Conformational dynamics of loop 102–112 and 210–220. (A and B) are the RMSD and RMSF for loop 102–112. (C and D) are the RMSD and RMSF for Thompson loop.

The atomistic deviations of <4.5 characterized the residues 102–112 and 211–220 were higher RMSD (∼6.5 Å) in the region of loop 40–54. Thompson loop was stable throughout while loop 102–112 deviated (2.0 to 4.5 Å). DMK9 and un-liganded *menG* systems had RMSF values of ∼4.25 Å and 4.00 Å respectively in the first loop (residues 40–54) and second (residues 102–112) loop while the DG70-bound complex had a higher RMSF value of ∼6 Å. Hence, the binding of DG70 was not felt around these loops although the RMSF significantly decrease to ∼3.2 Å in the second loop and remained lower (below 3.0 Å) in residues 211–220 than the RMSF of the Apo structure. This finding showed that Thompson loop has a profound impact in the binding of DG70 to *menG* and this correlates with the PRED which depicted that three of the interacting residues (GLY217, ILE219, and VAL220) found in the third loop.

Considering the averaged data, the DG70-bound *menG* had the highest mean RMSD value of 5.84 Å and mean RMSD values 3.34 Å in the loop 40–54 region. The mean RMSD value (5.84 Å) of Apo across the loops except for residues 102–112. Therefore, we can infer that the binding of DG70 caused instability in the *menG* structure as a result of inhibition.

Fig. 10C, 11B and D plots showed the RMSF values of the three loops. The highest average RMSF values of Apo, DMK9-bound, and DG70-bound were observed in the loop 40–54 region. In comparison, DG70-bound *menG* and Apo had RMSF values of 3.34 and 3.04, respectively, which implies similarities in their flexibilities. Table S1† (ESI) detailed the loops averaged RMSD and RMSF estimated for the unbound and bound-*menG* system.

**Fig. 12:**
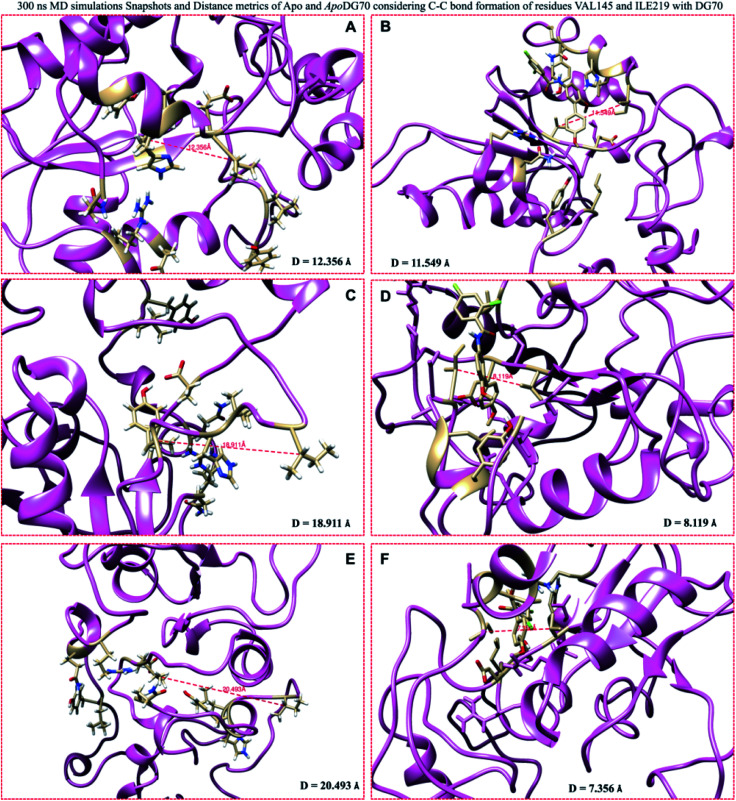
Distance (*D*) between VAL145 and ILE219 of the Apo and DG70-bound *menG* measured using the snapshots taken at 0, 150, 300 ns MD simulations. The *D* (Å) measured from Apo snapshots (ns) (A) (0), (C) (150), and (E) (300) are 12.356, 18.911, and 20.493 respectively. The *D* measured from DG70-bound snapshots (B), (D), and (F) are 11.549, 8.119, and 7.356, respectively.

To further probe into the inhibitory efficacy of DG70 on the inter-residual dynamics and motions of *menG*, the distance, *D* analysis was estimated from the snapshots of the 300 ns MD simulations of the Apo and bound-*menG* systems. The distances measured at 0, 150, and 300 ns between the interacting residues VAL145 and ILE219 of the bound and unbound *menG*. Fig. 12(A, C and E) showed that the distance between the two residues in the unbound *menG* decreases with increasing simulation time. At 0 ns, the distance was 12.356 Å and rose to 18.91 Å after 150 ns, and 20.49 Å after 300 ns. On the contrary, the distance between the two residues in the DG70 bound system decreases; between 0 ns and 150 ns, the distance decreased from 11.55 Å to 8.12 Å and again decreased to 7.36 Å after 300 ns Fig. 12(B, D and F).

The distance increased in the Apo structure and decreased in the DG70-bound in the same proportion as the simulation time. The decrease in the distance enhanced the DG70-bound *menG* interactions and the stability of the system. In other words, the decrease in the distance caused stronger interactions between the DG70 and the interacting residues at the active site, thus, promoting effective inhibition of the methylation activity of *menG* enzyme.

### 3.11 Secondary structure (DSSP) analysis of *menG* systems

A secondary structure assignment analysis carried out to study the prominent secondary structure elements, including alpha helices, beta sheets, loops, and coils in the *menG* protein during the whole 300 ns simulation time. There were 9α-helices, 4β-sheets, and three long conspicuous loops in the *menG* structure discussed above before simulations (ESI Fig. S3A†). However, after post-MD simulations, (ESI Fig. S3B†) revealed the visual transformations in the secondary structure of *menG* such as conformational changes in the helices and β-sheets, perhaps, changes to the loop structures because there seemed to be helices and beta sheets that transformed to loop.

Therefore, we did a comparative defined secondary structure of the un-liganded, DMK9-, and DG70-bound *menG* systems. The trajectories of the snapshots after at 300 ns were obtained and plotted using the in-house protocol. ESI Fig. S4† depicts the secondary structures of *menG* after 300 ns MD simulations. ESI Fig. S4A† is the unbound *menG* after 300 ns. We discovered that there were changes from the helices to loop and beta to loop after the 300 ns that led to the increased number of loops in the bound systems; there was a drastic change from alpha to bend (residues 1–101 and 142–178) and turn (residues 181–201) in DG70-bound *menG* (ESI Fig. S4C†) at 300 ns. There were transitions in the residues 31–39, 154–160, and 225–229 from the helices to bend upon DG70 binding. There were also transitions in the beta residues 85, 117, and 142–146 to bend. On the other hands, there was a change from alpha to bend upon the binding of DMK9 to *menG* (ESI Fig. S4B†) in residues 1–31 after the 300 ns. These changes in the elements of *menG* secondary structure were the cause of the instability^69^ that the DG70-bound *menG* displaced in the RMSF values of the whole and the loop regions stated in Sections 3.7 and 3.10.

## 4 Conclusion

The detailed molecular modeling study and the dynamics analyses provided in this report sets out the unique structural features and the conformational changes in *menG* whole structure and the omega-like loops especially the Thompson loop (residues 211–220) region. First, the Uniprot annotated residues were among the predicted active site residues. Nevertheless, the per-residue decomposition analysis showed that the none of the annotated residues did interact with DMK or DG70. On the other hand, PRED revealed that the substrate and the inhibitor bound to interacted with different residues which separates different active sites. Second, the predicted active site showed similarity to the previous prediction, however, the Ramachandran plot revealed that residues 7, 9, and 10 considered as interacting residues in the previous work are outliers.

Homology modeling revealed the secondary structure of the *menG* enzyme and some static features including the loops, α-helices, β-strands and GXG motif that showed similarities with the superfamily of methyltransferase. MD simulations showed motional deviations in the *menG* whole and its loop at the binding site. The stability of the DG70-bound system showed the consistency of the bound inhibitor at the active site throughout the 300 ns simulations. This complex stability correlates with the high binding free energy calculated and the residue–ligand interaction networks characterized by the strong hydrophobic, halogen, and hydrogen bond.

The graphical investigation of the loop dynamics at different time intervals confirmed the strong flexibilities that occurred in the whole structures of the free and the bound *menG* systems revealed by the RMSF analysis. The decreased distances between the interacting residues VAL145 and ILE219 in the DG70-bound system as against the increased distances between the same residues in the un-liganded system throughout a 300 ns MD further confirmed the binding impact of DG70 on inhibition of *menG* catalytic activity. The PCA eigenvectors obtained from the simulations showed clear variations among the systems which confirmed the dynamic conformational changes from the free to ligand-bound protein. Visually, there were some transitional changes in the secondary structure of *menG* to the loops hence, the free and the bound systems were analyzed using DSSP analysis which confirmed some conformational transformations such as helices to the loop.

Perhaps, it is important to carryout experimental analysis such as crystallographic studies to further explore the *menG* loops in relation to their functions in the ligand binding. The homology modeling insights will serve as aid to the researcher to synthesize the crystallized structure of this protein. Moreover, the demonstration of the above binding landscape of *menG* protein provides the basis that can utilize pharmacophore models in screening for better effective drugs with tolerable or no toxicity.

## Ethical approval

This article does not contain any studies with human participants or animals performed by any of the authors.

## Informed consent

This study did not require informed consent since the study does not contain any studies with human participants performed by any of the authors.

## Funding

No funding was received for this study.

## Conflicts of interest

The authors declare no conflict of interest.

## Supplementary Material

RA-010-D0RA03206A-s001
