# Supplementary material for: Thompson loop: opportunities for antitubercular drug design by targeting the weak spot in demethylmenaquinone methyltransferase protein
Source: RSC Adv. 2020 Jun 19;10(39):23466–83. doi: 10.1039/d0ra03206a (PMC9054810; doi:10.1039/d0ra03206a)
Supplement: RA-010-D0RA03206A-s001 [file RA-010-D0RA03206A-s001.pdf]

Electronic Supplementary Material (ESI) for RSC Advances.
This journal is © The Royal Society of Chemistry 2020

SUPPLEMENTARY

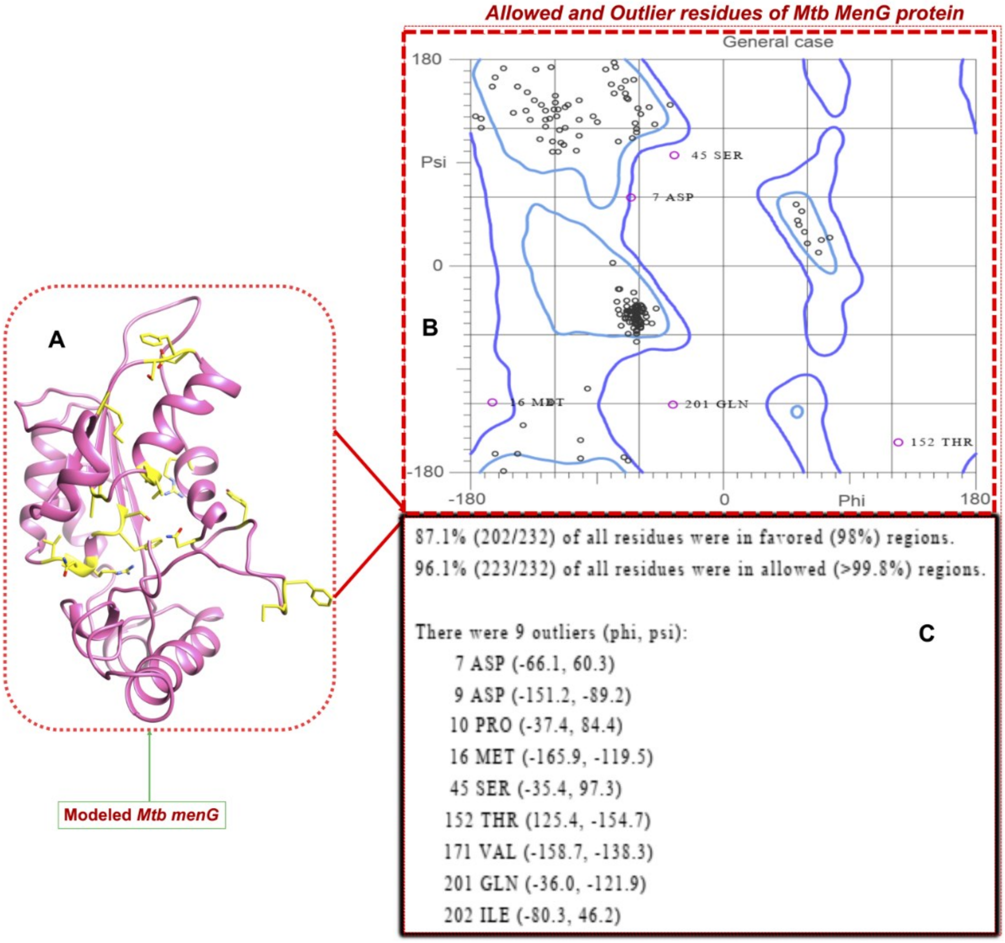

Figure S1. Ramachandran plot validation of the active site of the modeled *Mtb menG*.

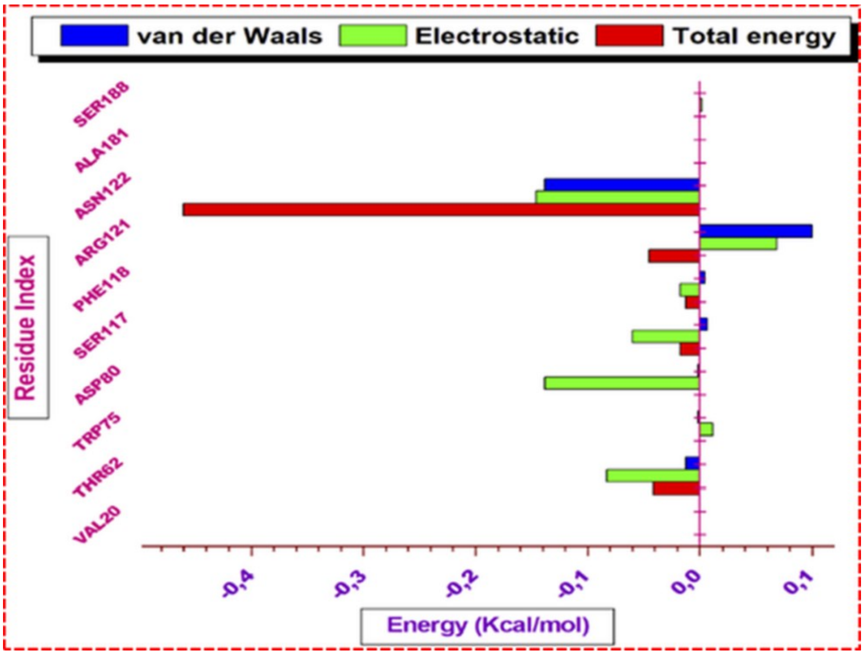

Figure S2. Per-residue decomposition of a *menG*-DG70 complex using the previous predicted active site that reported.

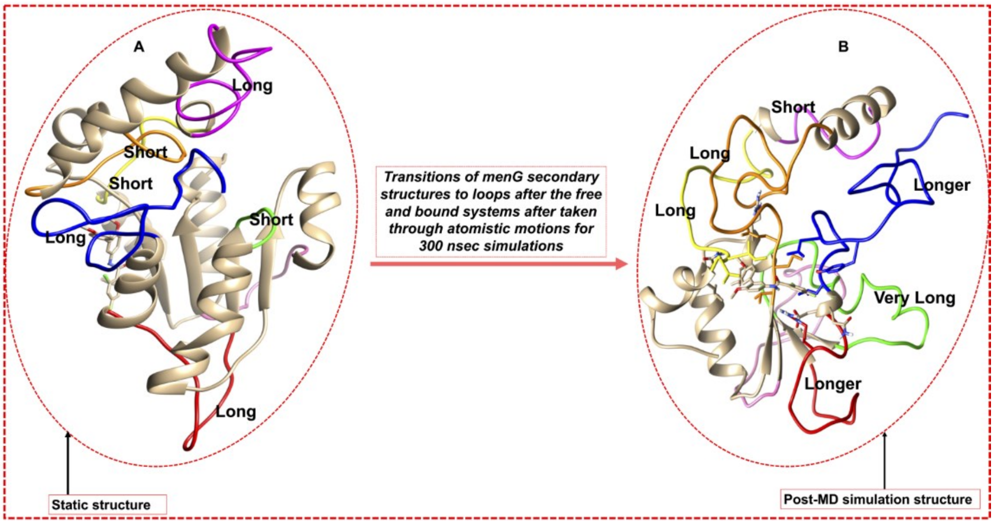

Figure S3 Secondary structure (α-helices and β-sheets) transformation to Loop structures over a 300 nsec trajectory.

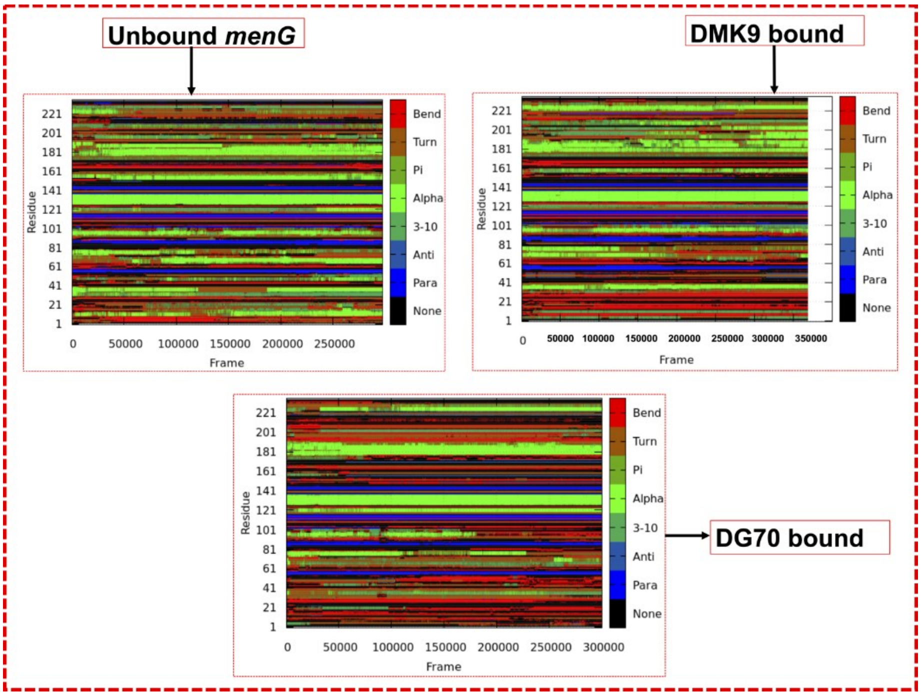

Figure S4. Illustrations of DSSP for *Apo* (**A**), ApoDMK9 (**B**), and ApoDG70 (**C**) systems over 300 nsec MD simulation.

Table S1. Mean RMSD and RMSF of the Apo, DMK9-, and DG70-bound

	Loop residues		
System	40-54	102-112	211-220
	Average RMSD (Å)		
Apo	5.26 ± 0.63	1.62 ± 0.17	4.77 ± 0.63
ApoDMK9	4.03 ± 0.84	1.60 ± 0.17	3.45 ± 0.30
ApoDG70	5.84 ± 1.03	2.69 ± 0.98	3.19 ± 0.43
	Average RMSF (Å)		
Apo	3.04 ± 0.80	1.32 ± 0.31	2.44 ± 0.67
ApoDMK9	2.86 ± 0.85	1.22 ± 0.23	1.29 ± 0.17
ApoDG70	3.34 ± 1.67	1.74 ± 0.69	2.07 ± 0.64
